# Clinical management of children with tic disorder: insights from therapeutic visits in China—a real-world study

**DOI:** 10.3389/fped.2024.1360470

**Published:** 2024-08-12

**Authors:** Jing Wang, Changyong Luo, Zhendong Wang, Tiegang Liu, Chen Bai, Yang Wang, Yuanshuo Tian, Qianqian Li, Zhaoxin Wang, Liqun Wu, Sumei Wang, Xiaohong Gu

**Affiliations:** ^1^ Pediatric Department, Wangjing Hospital of CACMS, Beijing, China; ^2^ Dongfang Hospital, Beijing University of Chinese Medicine, Beijing, China; ^3^ Gulou Hospital of Traditional Chinese Medicine of Beijing, Beijing, China; ^4^ School of Traditional Chinese Medicine, Beijing University of Chinese Medicine, Beijing, China; ^5^ Department of Chinese Medicine, Beijing Jishuitan Hospital, Capital Medical University, Beijing, China; ^6^ Dongzhimen Hospital Beijing University of Chinese Medicine, Beijing University of Chinese Medicine, Beijing, China

**Keywords:** tic disorder, real-world study, therapeutic medication, electronic medical data, traditional Chinese medicine

## Abstract

**Objective:**

This retrospective study aims to investigate the treatment of tic disorder (TD) in Dongfang Hospital affiliated with Beijing University of Chinese Medicine, explore its underlying mechanism, and provide valuable insights for future research and clinical management of TD.

**Methods:**

The electronic medical records of children with TD, from 2015 to 2021, were extracted from the information system of Dongfang Hospital affiliated with Beijing University of Chinese Medicine. The clinical characteristics of TD, utilization patterns of Chinese herbal medicine and synthetic drugs in prescriptions, as well as their pharmacological effects, were statistically described and categorized. In addition, association rules and network pharmacology were employed to identify core prescriptions (CPs) and elucidate their microscopic molecular mechanisms in treating TD.

**Results:**

The age range of the children was from 6 to 11 years, with a higher proportion of male participants than female ones. The average duration of treatment was 6 weeks. Regimen Z for the treatment of TD can be summarized as follows: Chinese herbal medicine [Saposhnikoviae Radix (FangFeng), Puerariae Lobatae Radix (GeGen), Uncariae Ramulus cum Uncis (GouTeng), Acori Tatarinowii Rhizoma (ShiChangPu), Chuanxiong Rhizoma (ChuanXiong)] and vitamins [lysine, inosite, and vitamin B12 oral solution] form the basic treatment, combined with immunomodulators, antibiotics, electrolyte-balancing agents, and antiallergic agents. CPs primarily exerted their effects through the modulation of gene expression (transcription), the immune system, and signal transduction pathways, with interleukin-4 and interleukin-13 pathways being particularly crucial. Among the lysine synthetic drugs used, inosite and vitamin B12 oral solution were the most frequently prescribed.

**Conclusion:**

The regimen Z drug treatment holds significant importance in the field, as it exerts its therapeutic effects through a multitude of pathways and intricate interventions. Chinese herbal medicine primarily regulates immune system–related pathways, while synthetic drugs predominantly consist of vitamins.

## 1 Background

Tic disorder (TD) is a neurodevelopmental disorder that has its onset in childhood and presents as motor and/or vocal tics (1). Its etiology may involve genetic, neurodevelopmental, psychological, and immune factors; however, research on TD is insufficient. The primary therapeutic goal is to reduce motor and vocal tics (2). Despite the publication of TD-related guideline recommendations by the 2021 European Society for the Study of Tourette Syndrome (ESSTS) and the 2019 American Academy of Neurology (AAN), there are variations in clinical practice regarding assessment, intervention, healthcare organization, patient preferences for pharmacological agents, availability and utilization of behavioral therapy, treatment costs, and approval status (1, 3–5). While some studies of the population characteristics of TD have been conducted in Asia through school surveys or epidemiological data sources (6), there remains a lack of such studies and specific treatments for children with TD obtained from real-world medical settings.

Although guidelines have recommended the use of traditional Chinese medicine (TCM) for the treatment of TD, there are some limitations. For instance, TCM products such as 5-Ling granule and Ningdong granule have been included in the list of compounds showing moderate confidence in the evidence of the effects of treatment according to the AAN guidelines. While TCM has been widely used in China for treating TD with positive outcomes (7), those who cannot read Chinese face difficulties in accessing effective information on the treatment of TD with TCM due to the fact that most studies are published in Chinese, as mentioned in the ESSTS guidelines.

Data mining can assist in identifying the correlations between herbs, while systematic pharmacology can help explore the mechanism of TCM prescription. TCM exhibits the characteristics of multielement and networked structure. A combination of the two approaches could provide support for doctors’ treatments and exploration of prescription mechanisms, which has been demonstrated in research on recurrent respiratory tract infections (8), chronic liver disease (9), *Mycoplasma pneumoniae* pneumonia (10), and coronary heart disease (11).

Therefore, based on real-world data on TD treatment, this study aims to analyze and summarize TCM treatments for TD through data mining and systematic pharmacology methods to broaden ideas and serve as a reference for future clinical research.

## 2 Materials and methods

### 2.1 Collection and processing of clinical data

#### 2.1.1 Data sources

The data were obtained from the visits of TD patients to Dongfang Hospital between 1 January 2015 and 30 September 2021.

#### 2.1.2 Database structure

The data from the information system of Dongfang Hospital was imported into Excel to establish a standardized structured database. The fundamental structure encompassed outpatient number, visit dates, birth dates, household registration locations, gender, diagnoses, prescriptions, etc.

#### 2.1.3 Data standardization

The medication information in the standardized structured database was further standardized by two researchers, respectively, with reference to the *Pharmacopoeia of the People's Republic of China* (12), Manual of Common Chinese and Western Medicine Drugs in Pediatrics (13), and the database of Chinese Medical Dictionary, and the standardized medicines were then classified according to the pharmacological effects of synthetic drugs.

### 2.2 Data analysis

#### 2.2.1 Analysis of basic information

Microsoft Office Excel 2019 was utilized for a statistical analysis of the basic information of patients and descriptive statistical analysis with regard to the frequency of synthetic drug usage and the use of herbs.

#### 2.2.2 The screening of herb core prescriptions

Prescriptions with more than three herbs were selected for further analysis using the R-Studio Apriori algorithm, a frequent item set algorithm that generated association rules that facilitated an examination of clear rules in TCM treatment practices. Each herb was regarded as an item set, while each prescription was treated as a transaction. Frequent item sets were identified to mine the association rules between herbs within prescriptions and obtain core prescriptions (CPs).

#### 2.2.3 Analysis of the therapeutic mechanisms of prescriptions

The molecular targets of the CP were obtained by querying the HERB database (http://herb.ac.cn/), while the disease targets were searched from both HERB and GeneCards databases (http://www.genecards.org/). A threshold of ≥1 was established in the GeneCards database to identify drug targets highly linked with diseases. The overlapping portion between drug targets and disease targets represented the one in which drugs acted on disease targets. A Kyoto Encyclopedia of Genes and Genomes (KEGG) pathway analysis utilizing Reactome (https://reactome.org/PathwayBrowser/#TOOL=AT) was performed, followed by plotting a KEGG pathway bubble map with the micro-bioinformation website (http://www.bioinformatics.com.cn).

## 3 Results

### 3.1 Clinical characteristics

The age and gender differences are provided in Tables 1 and 2. A total of 2,305 children with TD participated in the study, with 1,824 males and 481 females, resulting in a sex ratio of 3.8:1. Out of the total number of visits (13,949), males accounted for 11,215 visits, while females accounted for 2,734, yielding a sex ratio of 4.1:1. The number of visits for each child can be found in Supplementary Table S1. The mean number of visits for males and females was found to be approximately 6.15 compared with females, who had an average of approximately 5.68 visits per child. The regional distribution pattern is given in Table 3. Of the 2,305 children who enrolled, information on 1,797 household registrations was acquired and information on 508 registrations was missing. In geographical terms, the participants were spread across various provinces (30), municipalities, and autonomous regions within China.

**Table 1:** Age distribution of 2,305 children with TD.

Age	Number of patients	Percentage
1	1	0.04
2	8	0.35
3	22	0.95
4	84	3.64
5	177	7.68
6	403	17.48
7	310	13.45
8	330	14.32
9	246	10.67
10	218	9.46
11	145	6.29
12	113	4.90
13	84	3.64
14	68	2.95
15	30	1.30
16	32	1.39
17	24	1.04
18	10	0.43

**Table 2:** Age distribution of 13,949 children with TD.

Age	Number of cases	Percentage
1	1	0.01
2	10	0.07
3	62	0.44
4	293	2.10
5	741	5.31
6	2,058	14.75
7	1,725	12.37
8	1,863	13.36
9	1,644	11.79
10	1,563	11.21
11	1,165	8.35
12	840	6.02
13	625	4.48
14	531	3.81
15	332	2.38
16	248	1.78
17	164	1.18
18	84	0.60

**Table 3:** Regional distribution of 2,305 children with TD (ranked by percentage, top 10).

Region	Number of cases	Percentage
Beijing	888	49.42
Hebei Province	229	12.74
Shandong Province	116	6.46
Liaoning Province	67	3.73
Inner Mongolia Autonomous Region	60	3.34
Heilongjiang Province	60	3.34
Henan Province	52	2.89
Shanxi Province	51	2.84
Jiangsu Province	46	2.56
Jilin Province	35	1.95

### 3.2 Prescription

**Figure 1:**
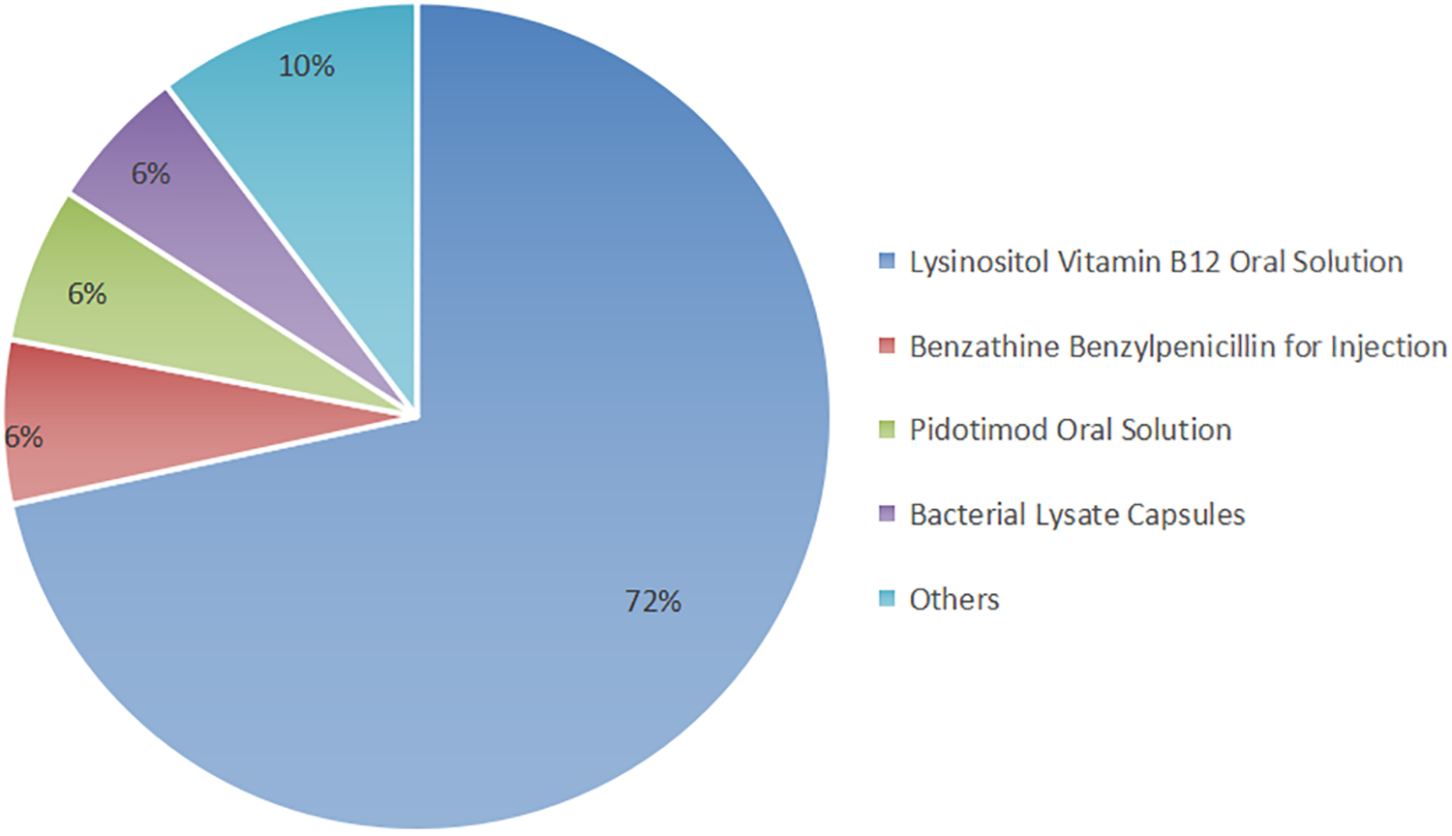
Distribution of the types of synthetic drugs.

**Figure 2:**
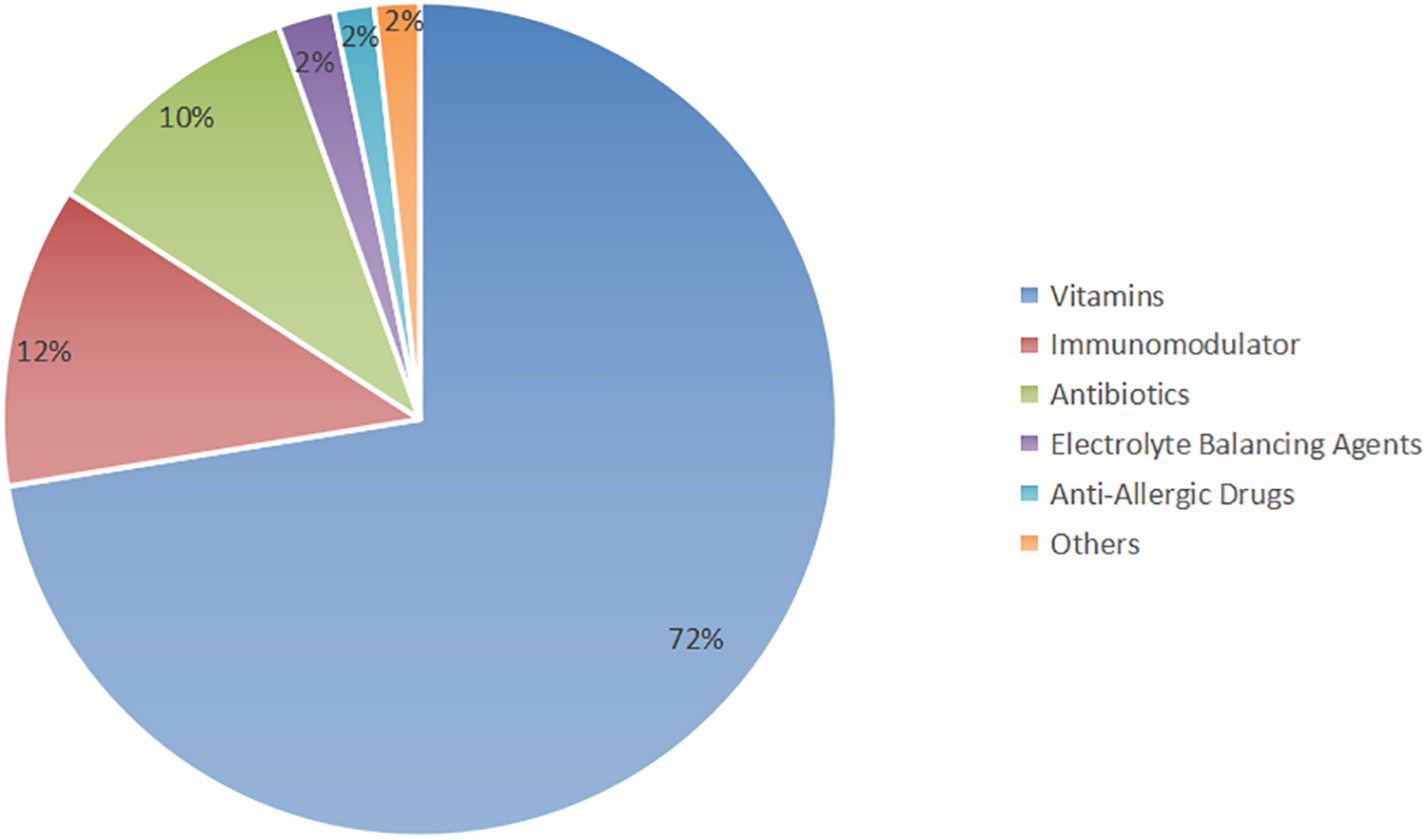
Distribution of the pharmacological effects of synthetic drugs.

The prescriptions consisted primarily of a combination of Chinese herbal medicine and synthetic drugs. The utilization of synthetic drugs is illustrated in Figure 1. Key synthetic drug components included lysine inosite and vitamin B12 oral solution, benzathine benzylpenicillin for injection, Pidotimod oral solution, and bacterial lysates, among others (refer to Supplementary Table S2 for detailed information). These drugs were categorized as vitamins, immunomodulators, antibiotics, electrolyte-balancing agents, antiallergic agents, and other classes based on their pharmacological effects; notably, the antiallergic agents were predominantly ophthalmic (Figure 2).

The 30 most frequently utilized herbs, accounting for 63.16% of the total herbs, are presented in Table 4. Among them, there were 14 herbs with a frequency proportion exceeding 2%: Saposhnikoviae Radix (FangFeng), Puerariae Lobatae Radix (GeGen), Uncariae Ramulus Cum Uncis (GouTeng), Acori Tatarinowii Rhizoma (ShiChangPu), Citri Reticulatae Pericarpium (ChenPi), Scorpio (QuanXie), Euricauli Flos (GuJingCao), Chuanxiong Rhizoma (ChuanXiong), Poria (FuLing), Pinelliae Rhizoma (BanXia), Gastrodiae Rhizoma (TianMa), Lycopodii Herba (ShenJinCao), Paeoniae Radix Alba (BaiShao), and Pheretima (DiLong).

**Table 4:** Distribution of commonly used Chinese herbal medicine (ranked by frequency, top 30).

Herbs	Total Frequency	Percentage
Saposhnikoviae Radix (FangFeng)	10,373	3.91
Puerariae Lobatae Radix (GeGen)	9,499	3.58
Ramulus Uncariae Cum Uncis (GouTeng)	8,918	3.36
Acori Tatarinowii Rhizoma (ShiChangPu)	8,359	3.15
Citri Reticulatae Pericarpium (ChenPi)	8,159	3.08
Scorpio (QuanXie)	7,647	2.88
Euricauli Flos (GuJingCao)	7,308	2.75
Chuanxiong Rhizoma (ChuanXiong)	7,294	2.75
Poriae (FuLing)	7,145	2.69
Pinelliae Rhizoma (BanXia)	7,059	2.66
Gastrodiae Rhizoma (TianMa)	7,009	2.64
Lycopodii Herba (ShenJinCao)	6,873	2.59
Paeoniae Radix Alba (BaiShao)	6,306	2.38
Pheretima (DiLong)	5,667	2.14
Bombyx Batryticatus (JiangCan)	5,077	1.91
Pseudostellariae Radix (TaiZiShen)	4,934	1.86
Zaocys (WuShaoShe)	4,769	1.80
Atractylodis Macrocephalae Rhizoma (BaiZhu)	4,760	1.79
Chaenomelis Fructus (MuGua)	4,563	1.72
Notopterygii Rhizoma et Radix (QiangHuo)	4,554	1.72
Dioscoreae Rhizoma (ShanYao)	4,068	1.53
Cinnamomi Ramulus (GuiZhi)	4,031	1.52
Bupleuri Radix (ChaiHu)	3,325	1.25
Bambusae Caulis in Taenias (ZhuRu)	3,236	1.22
Moutan Cortex (MuDanPi)	3,183	1.20
Angelicae Sinensis Radix (Danggui)	2,845	1.07
Prunellae Spica (XiaKuCao)	2,799	1.06
Rehmanniae Radix Preparata (ShuDiHuang)	2,672	1.01
Gardeniae Fructus (ZhiZi)	2,615	0.99
Platycodi Radix (JieGeng)	2,532	0.95

**Figure 3:**
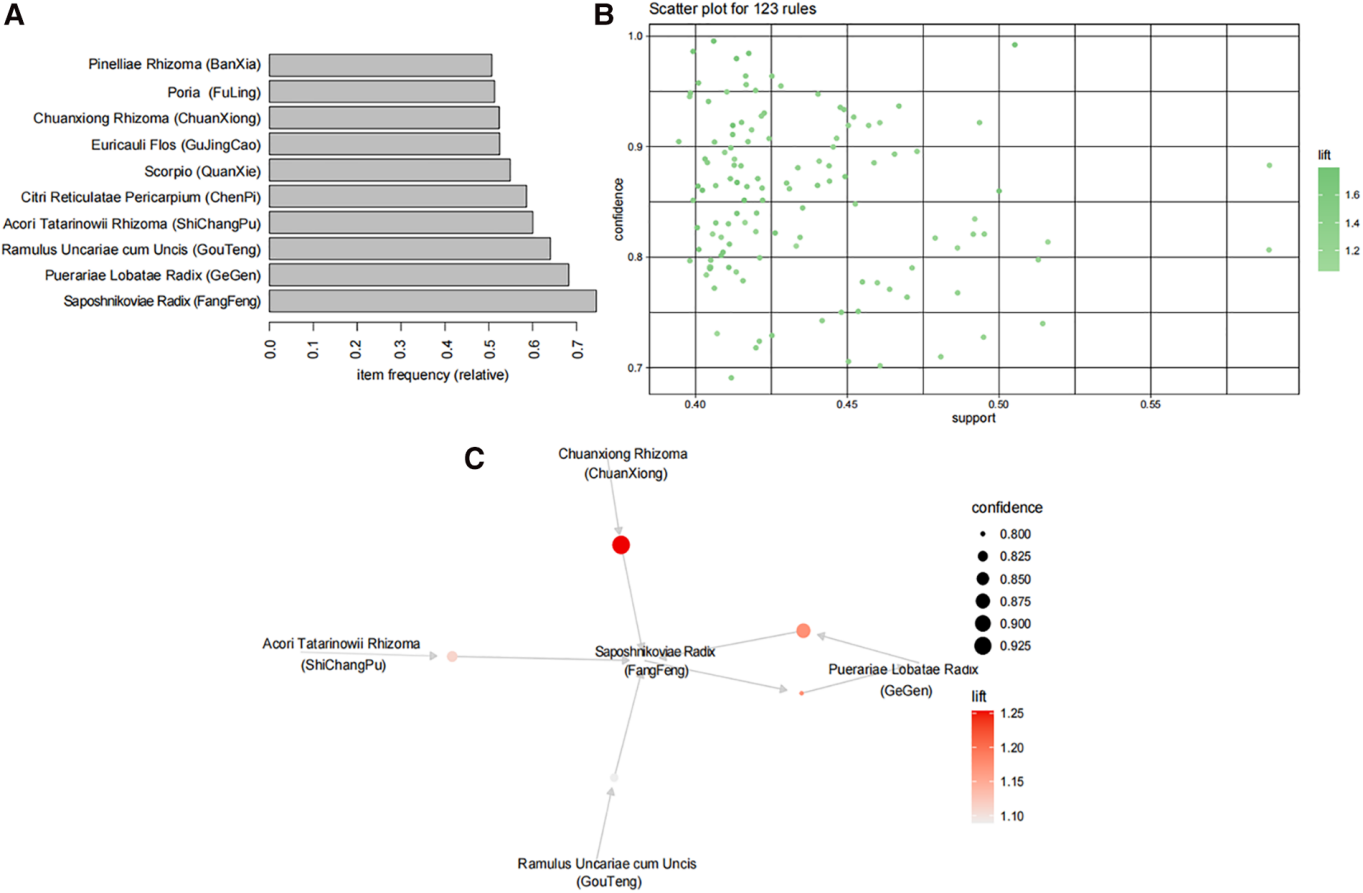
A CP analysis. **(A)** The top 10 highest frequency herbs are represented by the top 10 frequency items. **(B)** Association rules are depicted as dots, with a total of 123 rules showing the closest relationships between herbs. The x-axis represents support, while the y-axis represents confidence. The color of points indicates lift, with darker colors indicating higher lift values. **(C)** The core drug group association rule network; the top five support rules are shown in the figure. Each circle in the figure represents a rule, and its size corresponds to its level of support. Color is used to indicate lift value, with darker shades representing higher lift values.

The Apriori algorithm was employed to analyze the association rules among herbs in a total of 13,922 prescriptions containing more than three herbs each. First, the data were transformed by using transactions, and the top 10 frequently occurring items were identified as Saposhnikoviae Radix (FangFeng), Puerariae Lobatae Radix (GeGen), Ramulus Uncariae cum Uncis (GouTeng), Acori Tatarinowii Rhizoma (ShiChangPu), Citri Reticulatae Pericarpium (ChenPi), Scorpio (QuanXie), Euricauli Flos (GuJingCao), Chuanxiong Rhizoma (ChuanXiong), Poria (FuLing), and Pinelliae Rhizoma (BanXia), as shown in Figure 3A. By setting support = 0.4, confidence = 0.7, minlen = 2, and maxlen = 10, a total of 123 association rules were obtained as shown in Figure 3B (see Supplementary Data Sheet 1 for details of association rules). The top five support rules were selected for visual display (Figure 3C), which revealed that Saposhnikoviae Radix (FangFeng), Puerariae Lobatae Radix (GeGen), Ramulus Uncariae cum Uncis (GouTeng), Acori Tatarinowii Rhizoma (ShiChangPu), and Chuanxiong Rhizoma (ChuanXiong) exhibited strong correlations and occupied a core position within the prescription network denoted as CP.

From the HERB and the GeneCards databases, 342 drug targets of CP and 1,130 disease targets were obtained, which overlapped with bioinformatics and evolutionary genomics (http://bioinformatics.psb.ugent.be/cgi-bin/liste/Venn), resulting in the identification of 53 common targets (shown in Supplementary Table S3). This suggests that CP intervenes in TD through these 53 targets. Subsequently, a KEGG enrichment analysis was performed on these targets to identify important systems and pathways.

**Figure 4:**
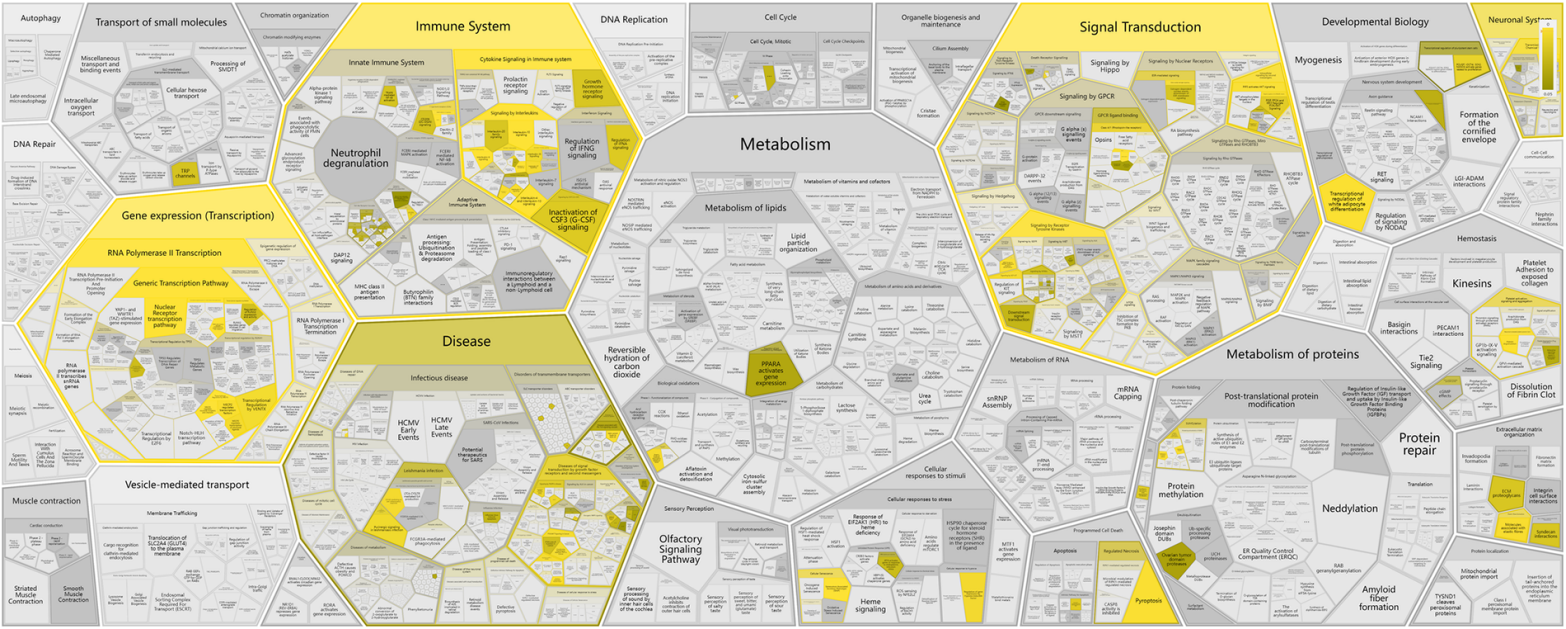
A genome-wide overview of CP in intervening TD. Higher brightness indicates a more significant correlation. Reprinted with permission from Milacic et al. (14), licensed under CC BY 4.0, https://doi.org/10.1093/nar/gkad1025.

The KEGG pathway analysis was conducted using the Reactome database, which revealed a close association between treatment with core Chinese herbal medicine and pathways related to gene expression (transcription), the immune system, and signal transduction (Figure 4).

**Figure 5:**
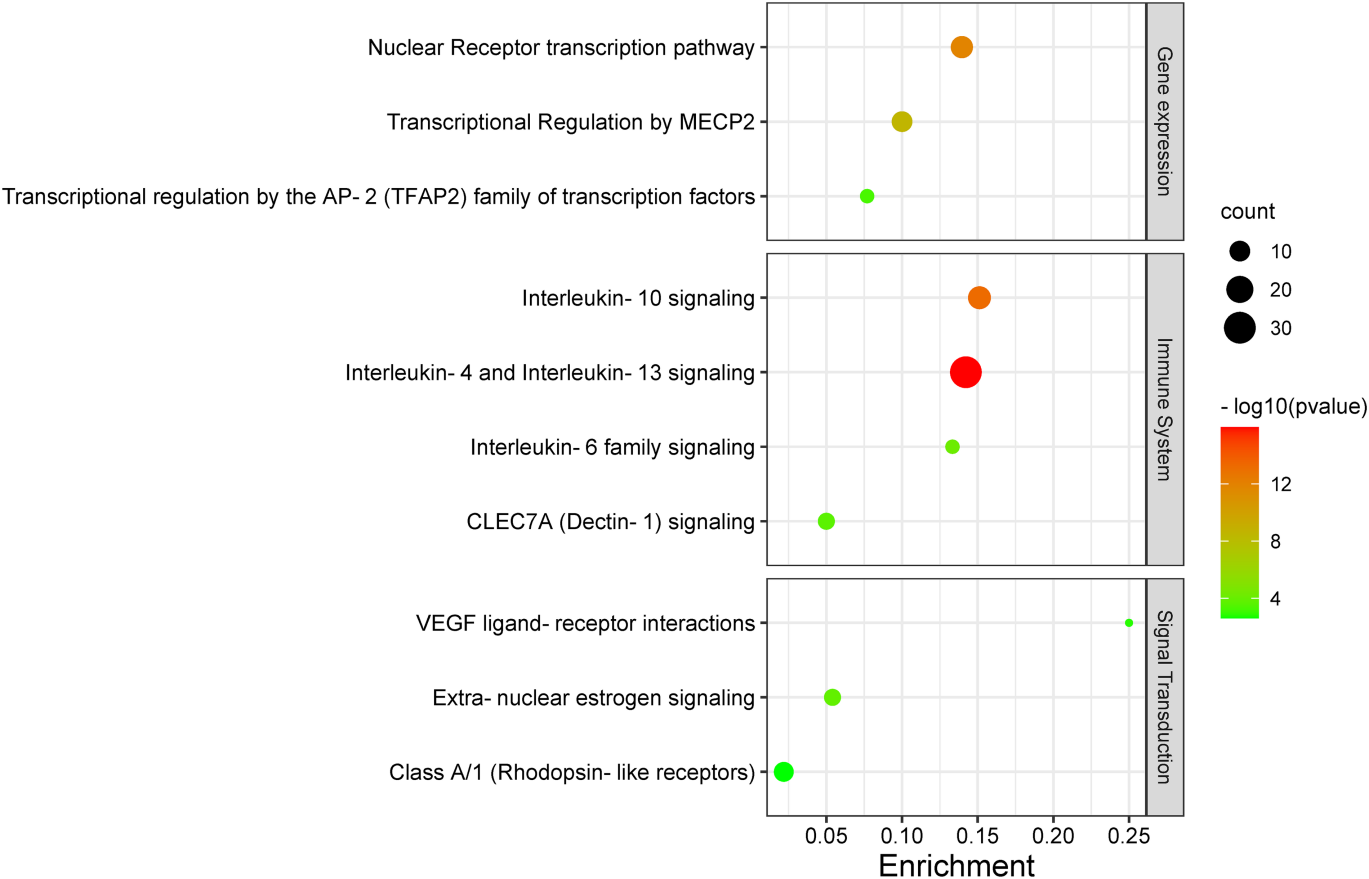
A KEGG enrichment analysis. Each bubble represents a distinct pathway, with the size of the bubbles positively correlated to the number of target genes within each pathway. Enrichment denotes the proportion of targets in relation to the total list, while larger bubbles indicate a greater richness of genes. The −log_10_ value represents statistical significance (*P*-value), where the redder color signifies smaller *P*-values and greater relevance to the respective pathway, whereas the greener color indicates larger *P*-values.

Figure 5 illustrates the key pathways influenced by this treatment, and these are as follows: the nuclear receptor transcription pathway; transcriptional regulation by MECP2 and by the AP-2 (TFAP2) family of immune transcription factor pathway in gene expression (transcription); interleukin-4 (IL-4) and interleukin-13 (IL-13) signaling, interleukin-10 signaling, interleukin-6 signaling, and CLEC7A (Dectin-1) signaling pathways in the immune system; extranuclear estrogen signaling, VEGF ligand–receptor interactions, and Class A/1 (Rhodopsin-like receptors) in signal transduction.

## 4 Discussion

The male to female ratio and the age characteristics of the children in this study were generally consistent with those reported previously (15), which may be attributed to the probability that TD in male children is more severe or complex than in their female counterparts, leading to increased medical intervention (16). This observation can be also explained by the developmental hypothesis of TD etiology (17–19). The manifestation of tic disorders in children is often influenced by the dynamics of parent–child relationships, peer interactions, academic pressures, and various other contributing factors. The peak presentation age at 6 years old is considered to be associated with factors such as school-related anxiety and situational changes, as well as the age of onset. In this study, a majority of children were recruited from 30 provinces, municipalities, and autonomous regions of Mainland China and some from Hong Kong, Macao, Taiwan, and foreign countries. This broad geographic representation suggested that the database was representative to some extent. It also showed that if environmental triggers were frequently involved in the onset of symptoms, they must be representative of all geographic areas covered in various studies (16). Although the etiology of TD remains unclear thus far, previous studies have indicated potential associations between TD and imbalances in trace element uptake, biological factors (immunity and neurobiochemistry), and psycho-psychological factors, as well as abnormal immune responses triggered by pathogens such as Streptococcus group A (6, 20–22). These findings could partially explain the effects of the synthetic drugs mentioned in this study. Notably, blinking was often observed as an initial symptom among visiting children and was commonly linked to allergic conjunctivitis (23), which could account for the high proportion of ophthalmic prescriptions identified within our study.

**Figure 6:**
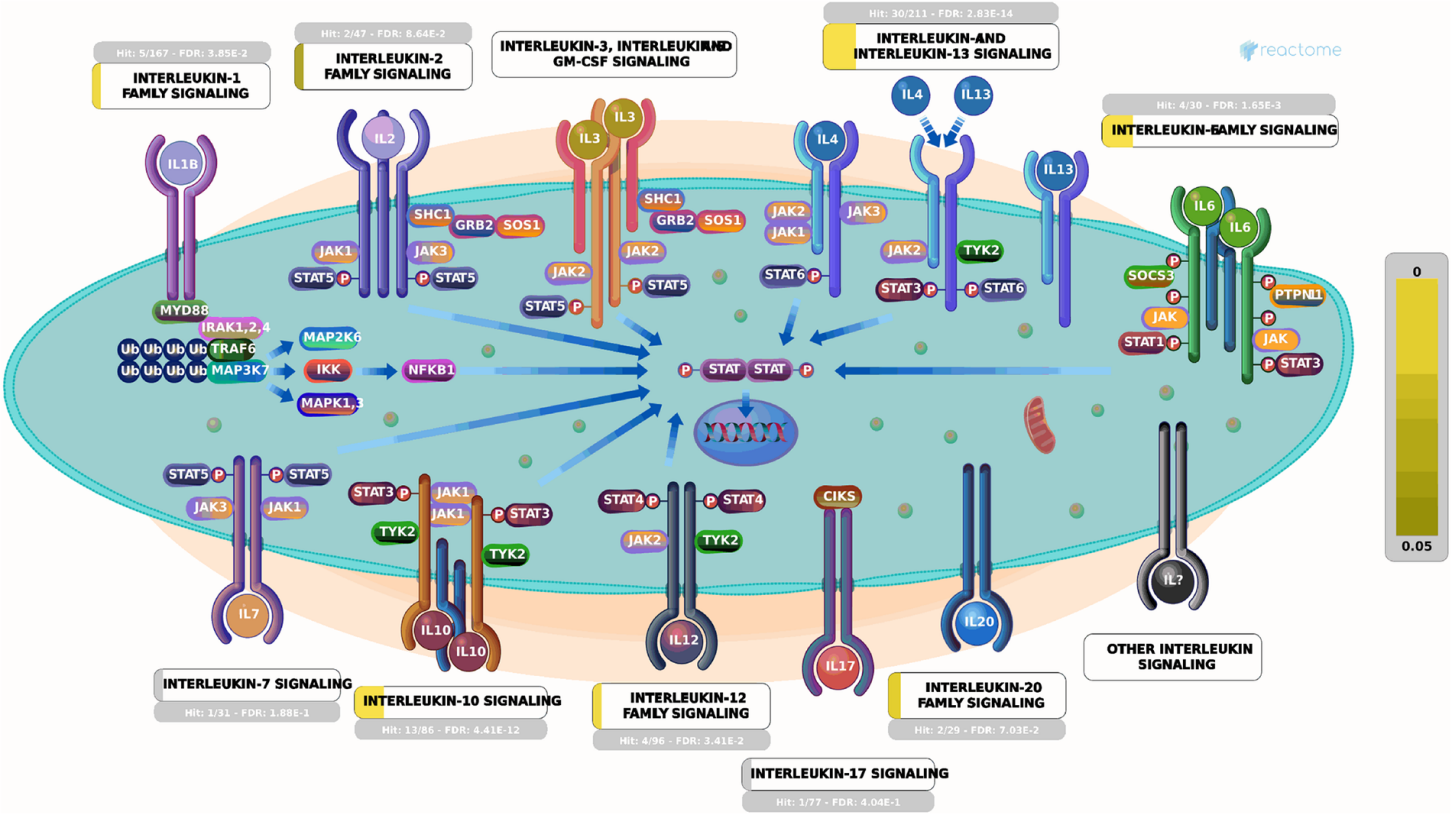
Signaling by interleukins. Each square represents a signaling. The larger proportion of yellow indicates that more genes are enriched. The brighter the color indicating smaller *P*-values, the more relevant it is to this pathway. Image Citation for Signaling by Interleukins. Reactome, 89, https://reactome.org/content/detail/R-HSA-449147 (July 08, 2024). Licensed under CC BY 4.0.

**Figure 7:**
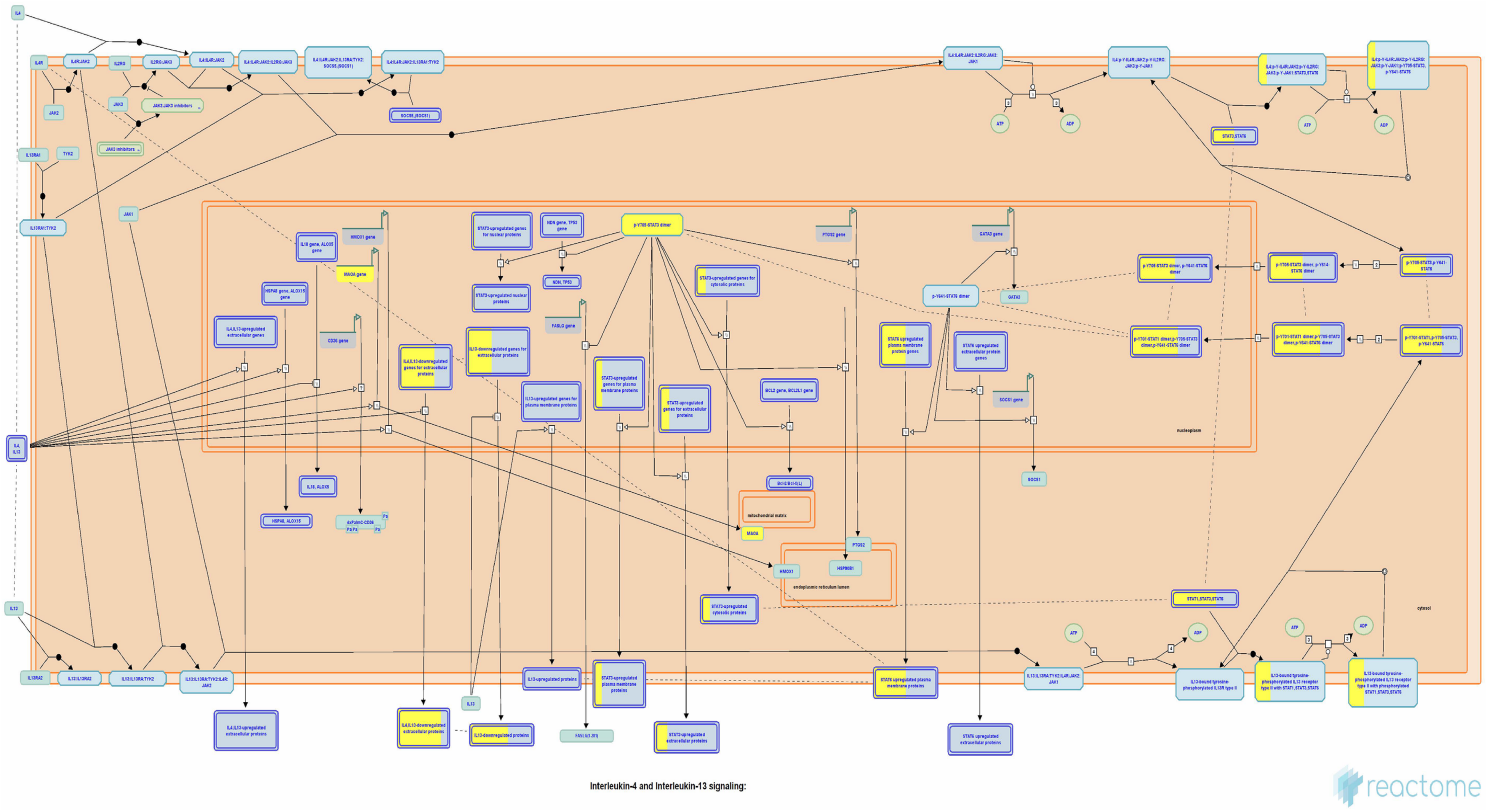
Interleukin-4 and interleukin-13 signaling pathways. Each square represents a signaling. The larger proportion of yellow indicates that more genes are enriched. Image Citation for Interleukin-4 and Interleukin-13 signaling. Reactome, 89, https://doi.org/10.3180/R-HSA-6785807.1 (July 08, 2024). Licensed under CC BY 4.0.

**Figure 8:**
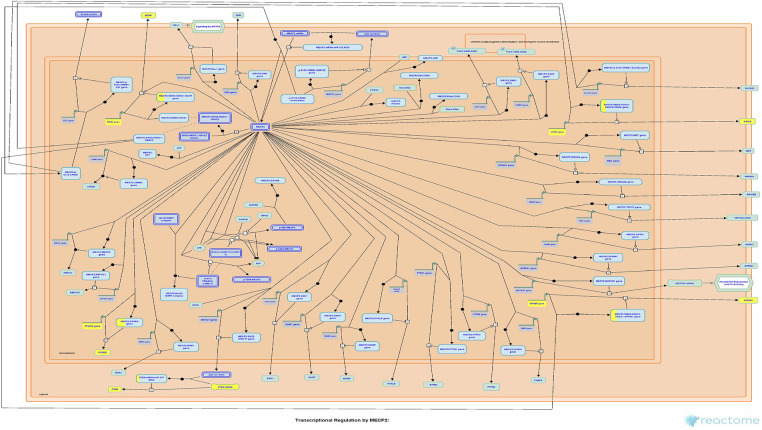
Transcriptional regulation by MECP2. Each square represents a signaling. The larger proportion of yellow indicates that more genes are enriched. Image Citation for Transcriptional Regulation by MECP2. Reactome, 89, R-HSA-8986944.1 (July 08, 2024). Licensed under CC BY 4.0.

Currently, there remains a lack of standardized treatment regimen for TD worldwide, and the overall efficacy of synthetic drug therapy is limited. Herbal medicine is considered an important alternative and complementary therapy to Western medicine, with TCM primarily focusing on controlling, relieving, and eliminating tic symptoms. Clinical treatment has demonstrated the effectiveness of CP according to TCM theory (24). This study aims to provide a more comprehensive basis for its clinical application by analyzing the potential mechanisms underlying its therapeutic effects on TD. The findings suggest that Chinese herbal medicine exerts a multichannel and complex intervention in treating TD. Notably, it exhibits significant effects onIL-4 and IL-13 signaling pathways within the immune system (Figures 6, 7). The severity of tic symptoms shows a negative correlation with IL-13, which displays anti-inflammatory properties and the ability to regulate immunoglobulin E (IgE) antibody production, and is negatively associated with the Yale Global Tic Severity Scale (YGTSS) scores (25–27). Murphy et al. observed elevated levels of anti-inflammatory cytokine IL-4 in patients on antibiotic and/or antipsychotic medications with tic symptom remission (28). Karagiannidis et al. (29) and Yael et al. (30) buttressed the findings of this study by demonstrating a significant correlation between transcriptional regulation by MECP2 (Figure 8) and TD. In this study, extranuclear estrogen signaling also shows a significant association with TD, suggesting that Chinese herbal medicine treatment may alleviate symptoms through the regulation of sex hormones. In addition, signaling pathways such as adrenoceptors, muscarinic acetylcholine receptors, and dopamine receptors exhibit significant differences in this study, indicating that Chinese herbal medicine treatment for TD may also intervene in neurotransmitters such as epinephrine, acetylcholine, and dopamine. Currently, researchers are focusing on dopamine regulation in TCM studies (31, 32). This study could expand our understanding of the mechanism underlying TD.

**Figure 9:**
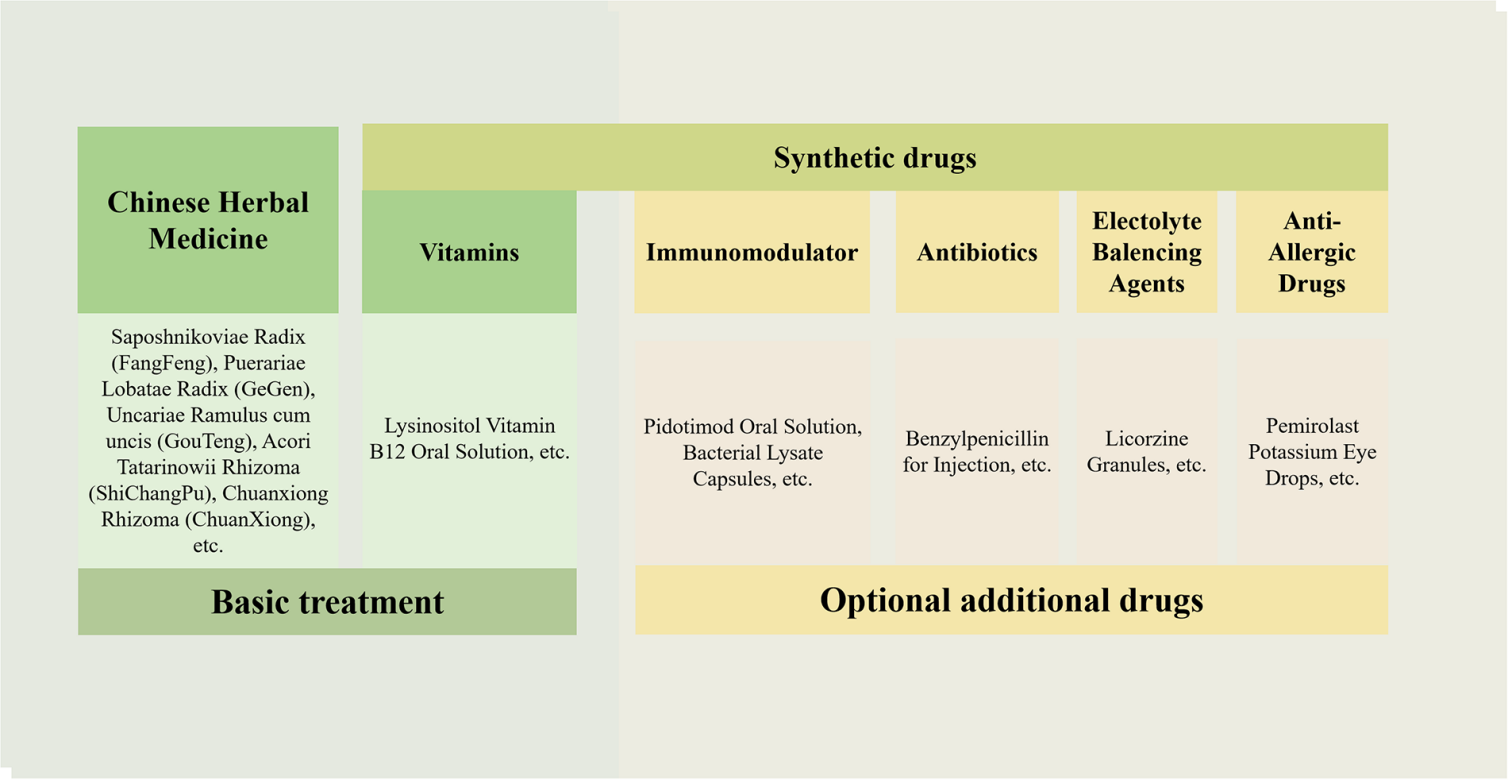
Regimen Z for TD treatment. The drugs used are mainly divided into traditional Chinese medicine and synthetic drugs. The treatment includes basic treatment and optional additional treatment.

It should be noted that the clinical characteristics revealed in the findings of this study were specific to patients with TD in the Department of Pediatrics at Dongfang Hospital. All patients expressed a preference for Chinese herbal medicine treatment, which is both legal and effective for TD in China (24, 33, 34). However, it is important to acknowledge that drugs such as rifampin, aripiprazole, tiapride, and clonidine were not included in the database of this study. This does not imply that these drugs were not used in the included patient cases. Based on extensive long-term clinical practice and a large sample size, the regimen for TD treatment (regimen Z) was summarized (Figure 9), which consists of two components: basic treatment and combined treatment. The basic treatment primarily involves Chinese herbal medicine and vitamins such as CP plus lysine inosite and vitamin B12 oral solution. Children with TD could be treated solely with this basic treatment or in combination with it, depending on their condition. Additional therapy options primarily include: first, immunomodulators such as Pidotimod oral solution, bacterial lysates, and so on; second, antibiotics such as benzathine benzylpenicillin for injection and so on; third, electrolyte-balancing agents, licorzinc, and so on; and fourth, antiallergic agents such as pemirolast potassium eye drops and so on. For children who have been regularly taking risperidone, aripiprazole, and other drugs, the general approach is outlined as follows:

First, maintain the original treatment regimen (regimen X), while adding regimen Z. Children with TD and immune dysregulation (e.g., recurrent respiratory tract infections) may receive additional immunomodulators such as Pidotimod oral solution or bacterial lysates, based on their condition. If streptococcal infection is suspected, combined antibiotic therapy is recommended. In case of electrolyte imbalance, combining electrolyte-balancing agents such as licorzinc may be considered. It was found in this study that there was a significant number of children with allergic conjunctivitis and other allergic diseases for which antiallergic agents such as pemirolast potassium eye drops could be applied.

Second, once regimen Z takes effect, gradually reduce the dosage of synthetic drugs in regimen X until they are discontinued, while continuing to adjust and use regimen Z. Third, continue using regimen Z accordingly. Finally, gradually decrease the dosage of drugs in regimen Z based on individual circumstances until complete cessation is achieved. Throughout the treatment process, healthcare professionals should provide strict medical advice to both children and their guardians emphasizing adherence. In addition, children's development is also influenced by contextual factors such as social relationships and academic pressures. In clinical practice, healthcare professionals appropriately address children's emotions based on the specific situation and provide guidance to their families. For instance, it is advisable to encourage families to maintain a harmonious attitude, refrain from criticizing their children's behavior, enhance family dynamics, and cultivate an environment that is conducive to the wellbeing of children. It is imperative to emphasize the importance of sufficient rest, adequate sleep, and limited exposure to stimulating activities.

Regimen Z could facilitate the understanding and application of TCM treatment options for TD, benefiting various stakeholders, including doctors, patients, and researchers. Based on a real and large electronic medical database, the characteristics of the real-world population of Chinese children with TD were analyzed in this study. Regimen Z predominantly employed Chinese herbal medicines such as Saposhnikoviae Radix (FangFeng), Puerariae Lobatae Radix (GeGen), Uncariae Ramulus Cum Uncis (GouTeng), Acori Tatarinowii Rhizoma (ShiChangPu), and Chuanxiong Rhizoma (ChuanXiong), along with vitamins such as lysine, inosite, and vitamin B12 oral solution, as fundamental treatment modalities. In addition, it incorporated immunomodulators, antibiotics, electrolyte-balancing agents, and antiallergic agents in combination therapy. The children received counseling services, while the families of the patients were provided with tailored professional advice to address their specific concerns. The findings of this study can serve as a valuable reference for future investigations and drug treatment strategies related to TD.

## 5 Innovation and limitations

To the best of our knowledge, this study represents the largest cohort of patients with TD treated with TCM to date. Patients were recruited from across the country and received treatment at Grade A hospitals under authoritative guidance. Through an analysis of a large sample size, we identified key drug therapeutic strategies and explored their underlying mechanisms, providing valuable insights for future research in TD treatment.

The limitations of this study were the lack of information on some registered residences in the database. In addition, although acupuncture (35), massage, press needle (36), behavioral intervention, family support, and other treatment modalities were commonly used for pediatric TD at Dongfang Hospital alongside Chinese herbal medicine and synthetic drugs, they were not comprehensively included in this study because of the lack of data sources and other constraints. The study was centered on pharmacotherapeutic strategies. In addition, China has a vast territory, and the samples were mainly obtained from Northern China. Adapting to local conditions is the basic principle of TCM treatment. Hence, the treatment regimen may not be appropriate for Southern China where local conditions differ. Moreover, the results might demonstrate bias because of the absence of large-scale, multicenter, randomized controlled trial. Nevertheless, it is worth mentioning that the prescriptions originated from a prestigious third-class Grade A hospital in Beijing, which is considered an authority in Chinese pediatric medicine. The clinical practice insights derived from data mining are highly valuable for professionals. Future research should incorporate diverse methodologies such as drug safety testing, scalability evaluation, comorbidity analysis, behavioral cognitive therapy, and other approaches to enhance the diversity of data. Furthermore, a prospective multicenter, randomized, double-blind controlled trial must be conducted to comprehensively assess prescriptions. Further experimental studies are also warranted to elucidate and validate the mechanisms of action underlying these prescriptions.

## Data Availability

The original contributions presented in the study are included in the article/**Supplementary Material**, further inquiries can be directed to the corresponding authors.

## Supplementary material

Supplementary Table S1 Number of visits.

Supplementary Table S2 The type and quantity of synthetic drugs.

Supplementary Table S3 Target information.

Supplementary Data Sheet 1 The important association rules of the prescriptions of the tic disorder were obtained using the Apriori algorithm.
